# The Pathology of Insanity

**Published:** 1850-10-01

**Authors:** John Hitchman


### THE PATHOLOGY OF INSANITY.

A LECTURE, DELIVERED MAY 25TH, 1850, IN THE MIDDLESEX COUNTY ASYLUM, HANWELL,

BY DR. JOHN HITCHMAN.

GENTLEMEN,—The time has now arrived, in which it becomes my duty to solicit your attention to some "dry statistics," and to facts which, as they have no immediate practical bearing, may, I fear, prove somewhat tedious to you. The portion of labour allotted to me, is to describe the appearances found after death in the bodies of the insane, and to inform you of the conclusions to which I have come respecting them.

In the great hospitals of London, the various derangements of the body have attracted your attention. You have studied the disorders of the ear, and investigated the diseases of the eye:—you have learned to detect, to diagnose, and to treat the various injuries to which the arm or the leg may be exposed, and to amputate the injured member when all remedial resources have failed to heal. You have studied the varied functions of the body, and have learnt such remedies as are calculated to bring back health and vigour to the limbs which have been rendered powerless by disease. I rejoice that you have proceeded further, and that you have, in the wards of this great institution, directed your attention to the diseases which affect the mind. For in comparison with these, all other ailments sink into insignificance; thus the convulsive twitchings, the short vision, and the endless aches which Johnson endured were considered by him as trifles, compared with one dreadful disease which seemed to threaten him. "Of the uncertainties of our present state," he says, "the most dreadful and alarming is the uncertain continuance of reason." All reflecting minds will agree with the great moralist on the transcendent importance of this subject. The disorders of the senses, of the limbs, of the body, possess some mitigating circumstances. Deafness, total and complete, may beset a man: the voice of friendship, the tones of childhood, or the language of love, may never fall upon his ear; his life may be passed amid unbroken silence, and yet, like Kitto, he may enrich the biblical literature of his country, and be useful to all generations. A man may be blind, and yet pour forth from the treasure-house of his soul such truths as the "world will not willingly let die." Physical darkness may surround him, and yet he may perceive the lovely in nature, and the beautiful in art. The virtue, the wisdom, the valour, and the beauty of Greece and Troy may move before him, and their heroes be rendered deathless by his own narration of their glorious deeds. A man may be blind, and yet the gales of Paradise may fan his brow—the rich discourse of angels fall upon his ear, and

> "Adam, the goodliest man of men since born,
> His sons; the fairest of her daughters, Eve"

may walk within his view: for him

"The morn, with rosy steps, may sow
The earth with orient pearl,"

and in the consciousness of duties nobly done, he may triumphantly exclaim—

"It is not miserable to be blind.
———— Nor bate a jot of heart, or hope,
But still bear up, and steer right onwards."

A man may lose an eye and an arm, and yet do the "state some service." From the proud waves of Trafalgar he may raise the shout of victory, and telegraph a sentiment that shall be lasting as the language in which it was spoken. The limbs may be powerless; but if the mind be firm, the patriotic statesman, like Chatham, may be usefully carried to the council-chamber of his country, and there, with indignant eloquence, as with the peal of a war-trumpet, or the blast of the thunder-storm, he may startle the lethargy and awake the patriotism of the indolent and the careless. But if the mind be diseased, then—"Farewell to all his greatness"—the man is useful no more. His best friends wish to hide him from the gaze of the world, and even sympathy for his fate will sometimes die away. His whole being is changed—nay, his very virtues are tarnished. The brave man dwindles to a coward; the wit becomes a fool—

"From Marlboro's eyes the tears of dotage flow,
And Swift expires a driveller, and a show."

Surely here is a mission high enough for the greatest amongst us. Can medicine have a loftier aim than to alter this state of things? to call back the troubled mind to health, to peace, and to usefulness. Are you philosophers, as well as medical men? do you delight to exercise your minds in abstruse speculations, to investigate the nature of things, to study *physiology* in the high and legitimate sense of that term? Here, too, this subject has its charms. The nature and the attributes of the human mind have engaged the attention, and absorbed the faculties of some of the wisest of our race. It is a study which attracted alike the philosophers of Egypt, Greece, and Rome; and which, outstripping in the grandeur and intensity of its interest all that even the most sublime of the physical sciences had to bestow, could make Plato thus apostrophise his own being—

*’Αστέρας εἰσαθρεῖς, ἀστὴρ ἐμός· εἴθε γενοίμην
οὐρανὸς, ὡς πολλοῖς ὄμμασιν εἰς σε βλέπω.*

The magnificence of the "starry sky," even when illuminated by that light, which Newton revealed to mankind, even when "cycle in epicycle, orb in orb," and world after world roll before the eye, in all the majesty and splendour and beauty of harmonious law, pales before the still brighter marvel of the human mind; which, after recognising the beauty of the celestial bodies, measuring their magnitudes, discovering their laws, and predicating their movements, can rise from the great perception of a law, to the still greater idea of a Lawgiver. No wonder, then, that this great power should have attracted notice in every age; that its nature should have been closely scrutinized, and its attributes carefully recorded. Nor need we be surprised that it continues to be imperfectly known; for the march of truth is as slow as it is majestic. It has ever been so. The circulation of the blood had been suggested by the discoveries of Cesalpinus, Columbus, and Servetus, before the time of Harvey. Vaccination, as a prophylactic agent against small-pox, had been long talked of by the Gloucestershire peasantry, before it was practically enforced by Jenner. Steam had been experimented upon, and its expansive power detected, by the Marquis of Worcester, a hundred years before it was brought into the full service of mankind by the talents and skill of James Watt. Unzer, Prochaska, and Whytt, had long groped among the facts, which in our own day have moulded themselves into a marvellous and beautiful "*diastaltic nervous system*," under the plastic genius of Marshall Hall; thus, as the Poet Tennyson has quaintly expressed it—

> "Through the ages, one increasing purpose runs,
> And the thoughts of men are widened by the process of the suns."

The deep meditations of Thales within the beautiful islands of the Ionian Seas, and the philosophic speculations of Pythagoras respecting the human soul, led on to the sounder theories of Parmenides and the Eleatics, and through them ultimately to the school of Socrates and Plato. From the genius of Plato arose the neo-platonic philosophy, and the doctrines of the Alexandrian school. The inadequate and speculative character of the ancient metaphysical philosophy aroused (in the course of ages) the master-minds of Des Cartes and Bacon, by whom the "inductive method" was revealed for the improvement and guidance of mankind.

In passing over the metaphysical theories, which for several centuries engrossed the attention of reflecting minds; in reviewing the utterances of these ancient philosophers by the light of the present age, one is perfectly astonished at many of their statements;—thus Pythagoras could recognise in the human soul three distinct faculties, reason (*νους*), intelligence (*φρὴν*), and desire (*θυμὸς*); regarding the first as peculiar to man, while the latter two were endowments which he possessed in common with all animals: and Parmenides, who flourished five hundred years before the Christian era, actually gave utterance to the great principle, which has been in our own time so ably supported and enforced by Gall and Spurzheim—Parmenides said, "the highest degree of organization gives the highest degree of thought."

This, with certain restrictions, is undoubtedly true, but mankind have ever loved extremes. In religion, in politics, and even in philosophy, this has been universal. The French encyclopedists, recognising the variety and the grandeur of physical facts, and perceiving that we become acquainted with objective nature in all her countless forms through the agency of the senses, originated that "sensational philosophy," which was so warmly espoused, and ably advocated, by our immortal countryman, John Locke. The genius of Berkeley, penetrating the surface of things, feeling assured that phenomena must have a substance, a "noumena" for its very existence and manifestation, that, in truth, the senses could only detect the quality, or property, rather than the substance of things, and that there were in man volition and impulses innate, and independent of all sensation; and knowing further, that the information conveyed by the senses required the correcting influence of the pure reason, regarded *all* such information as treacherous, and wholly dependent for its form upon the subjective powers of the human mind, thus becoming as extreme in one pole of thought as Cabanis or Gall was at the other—if, indeed, Fichte of Germany has not surpassed him in the transcendental notions of the same idea. Let it be ours to adopt a spirit of eclecticism from these various schools, avoiding the extreme materialism of the one, and the speculative idealism of the other;—knowing, as we do from experience, that by some mysterious and inexplicable union matter is influenced by mind, while mind is dependent for its manifestations upon the healthy condition of the material brain. In psychology, as in physics, it is well to take Bacon for our guide, who says—

"Duæ viæ sunt, atque esse possunt ad inquirendam et inveniendam veritatem. Altera a sensu et particularibus advolat ad axiomata maxime generalia, atque ex iis principiis eorumque immota veritate judicat et invenit axiomata media: atque hæc via in usu est. Altera à sensu et particularibus excitat axiomata ascendendo *continenter* et *gradatim*, ut ultimo loco perveniatur ad maxime generalia, quæ via vera est, sed intentata."

In the lecture which I gave in this room last year, and which you will find published in the current number of the Psychological Journal, thirty seven cases were selected for observation: now, however, I purpose embracing a larger amount of time, and to include the deaths of four years, which will enable us more nearly to conform to the Baconian precept—by having a larger basis of facts, from which to proceed "continenter et gradatim." The following synopsis embraces all the particulars:—

*From January* 1*st,* 1846, *to January* 1*st,* 1850, *inclusive, there have died* 103 *Female Patients: of these, the respective ages were as follow:—*

| | |
|---|---|
| From 20 to 25 years | 2 |
| „ 25 to 30 do. | 6 |
| „ 30 to 35 do. | 9 |
| „ 35 to 40 do. | 8 |
| „ 40 to 45 do. | 5 |
| „ 45 to 50 do. | 13 |
| „ 50 to 55 do. | 7 |
| „ 55 to 60 do. | 5 |
| „ 60 to 65 do. | 12 |
| „ 65 to 70 do. | 8 |
| „ 70 to 75 do. | 11 |
| „ 75 to 80 do. | 5 |
| „ 80 to 90 do. | 1 |
| Not ascertained | 11 |
| Total | 103 |

*Causes of the* 103 *Deaths, from January* 1*st,* 1846, *to January* 1*st,* 1850, *inclusive.*

DISEASES OF THE BRAIN AND ITS MEMBRANES.

| | |
|---|---|
| Exhaustion after mania | 5 |
| Apoplexy | 6 |
| ,, serous | 2 |
| Epilepsy | 4 |
| General paralysis | 11 |
| Paralysis | 3 |
| Softening of the brain | 3 |
| Fungoid tumour of the dura mater | 1 |
| Fracture of the skull and extravasation of blood, from an accidental fall | 1 |
| Total | 36 |

DISEASES OF THE RESPIRATORY ORGANS.

| | |
|---|---|
| Pulmonary consumption | 19 |
| Pneumonia, and Hepatization of lung | 4 |
| Abscess of the lung | 3 |
| Emphysema of lung | 1 |
| Pulmonary apoplexy | 1 |
| Hydrothorax | 1 |
| Chronic bronchitis | 1 |
| Total | 30 |

DISEASES OF THE HEART, AND ITS MEMBRANES.

| | |
|---|---|
| Pericarditis | 1 |
| Hypertrophy of the heart | 2 |
| ,, with Anasarca | 1 |
| Fatty degeneration of the heart | 1 |
| Total | 5 |

DISEASES OF THE DIGESTIVE ORGANS, AND THEIR CONNEXIONS.

| | |
|---|---|
| Scirrhus of liver | 1 |
| Cirrhosis of liver | 1 |
| Inflammation of bowels | 2 |
| Dysentery | 1 |
| Total | 5 |

DISEASE OF THE URINARY ORGANS.

| | |
|---|---|
| "Bright's Disease" of the kidney | 1 |

DISEASES OF THE REPRODUCTIVE ORGANS.

| | |
|---|---|
| Scirrhus of ovaries | 1 |
| ,, and abscess of do. | 1 |
| ,, of uterus | 4 |
| Total | 6 |

CONSTITUTIONAL DISEASES.

| | |
|---|---|
| General debility | 11 |
| ,, and abscess of back | 1 |
| Exhaustion from abscess | 1 |
| Old age and natural decay | 5 |
| Total | 18 |

| | |
|---|---|
| Cancer of mamma | 1 |
| Erysipelas | 1 |
| | 2 |
| Total | 103 |

*Form of the Mental Malady in the* 103 *Patients, who have died between January* 1*st*, 1846, *and January* 1*st*, 1850, *inclusive.*

| | |
|---|---|
| Mania | 20 |
| „ with epilepsy | 1 |
| „ with general paralysis | 4 |
| Melancholia | 4 |
| „ with general paralysis | 3 |
| Incoherence | 13 |
| Imbecility | 34 |
| „ with epilepsy | 7 |
| „ general paralysis | 1 |
| Dementia | 5 |
| „ with epilepsy | 3 |
| „ and general paralysis | 1 |
| „ general paralysis | 2 |
| Senile insanity | 4 |
| Idiocy, with epilepsy | 1 |
| | 103 |

*The Duration of the Mental Malady, in these* 103 *Patients, was as follows:—*

| | |
|---|---|
| Not more than 6 months | 4 |
| „ „ 1 year | 1 |
| „ „ 2 do. | 6 |
| „ „ 3 do. | 8 |
| „ „ 4 do. | 4 |
| „ „ 5 do. | 5 |
| „ „ 6 do. | 2 |
| „ „ 7 do. | 5 |
| „ „ 8 do. | 2 |
| „ „ 9 do. | 5 |
| „ „ 10 do. | 1 |
| „ „ 11 do. | 3 |
| „ „ 12 do. | 1 |
| „ „ 13 do. | 1 |
| „ „ 14 do. | 3 |
| „ „ 15 do. | 0 |
| „ „ 16 do. | 0 |
| „ „ 17 do. | 2 |
| „ „ 18 do. | 3 |
| „ „ 19 do. | 3 |
| „ „ 20 do. | 2 |
| For more than 20 years | 18 |
| Not ascertained | 24 |
| Total | 103 |

Of these patients, ninety-four were submitted to post-mortem examinations. In fifty-one of these, the calvarium presented some slight departure from healthy structure—this being chiefly absorption, or attenuation in various parts. The dura-mater was preternaturally adherent in about one-third of the cases. In nine, the superior longitudinal sinus was thickened by long deposits. In thirty-seven, and these chiefly among the aged, the basilar, and cerebral arteries were atheromatous. In four, there were tumours, varying in size from that of a hen's egg to a walnut, arising from the dura-mater, and pressing upon the anterior, lateral, and superior surfaces of the cerebral lobes. In ninety, the arachnoid membrane was more or less opaque, thickened, or inflamed; in a corresponding number, the pia-mater was congested with blood, or infiltrated with fluid, and in one case, (an epileptic,) it was strewed with hydatids. In sixty-eight cases, there was marked shrinking, or atrophy of the convolutions, and in all these, there was an excess of sub-arachnoid fluid. The cineritious neurine of the convolutions was injected, and of a rose-colour in three cases, all of which were recent, and died while suffering under acute mania. This structure was pale and blanched in sixty-four instances; it was of a dark brown colour in six instances. The fibrous parts of the encephalon were softened in eleven cases, and greatly congested in twelve cases; in five cases, (and all these died from general paralysis,) it was firm, tough, and granulated along the surface of the lateral ventricles. The thalami optici and the striated bodies, as well as the corpus callosum, were softened in three instances, in which the general structure of the encephalon did not participate in the malady, and in all these there was paralysis prior to death, of a paraplegic or hemiplegic character. In three cases, there were great differences in the size of the opposite hemispheres of the brain; in one, the left lobe was one-third less than the right, and the whole brain weighed only one pound, six ounces and a half. This patient was idiotic, and the right arm was shrunken and paralysed from birth. In ten cases, the lateral ventricles were stretched, and distended with clear serous fluid; and in two patients, who died of apoplexy, the right ventricle was filled and lacerated by effused blood: in one patient, the middle of the left hemisphere was distended with pus. Thus, in ninety-three cases, there was disease, of some kind or other, in the cranium, the membranes, or the brain, and in one only, was there no trace of cerebral disease, and she died of cirrhosis of the liver, after having been for some time despondent and suicidal; and I may add, that in the examination of nearly 400 cases, (with the exception of the above case,) I have invariably found some change in the structure of the brain or its covering. Other institutions, where cases in *an early stage* of the malady are examined, have published very different results; thus, Dr. Webster has given the summary of 165 cases examined at Bethlem Hospital, as follows:—

| | |
|---|---|
| The arachnoid membrane thickened and opaque in . . . . . | 62 |
| The pia mater infiltrated in . . . . . . . . . . . . . . . | 145 |
| Turgidity of the blood-vessels in . . . . . . . . . . . . . | 120 |
| The colour of the brain was altered from its natural tint in . . . | 45 |
| Blood was effused in . . . . . . . . . . . . . . . . . | 32 |
| And the medullary parts were congested in . . . . . . . . . | 32* |

A microscope consisting of a simple lens, or doublet, frequently reveals changes of structure which pass unobserved by the unassisted eye, and

* Medico-Chirurgical Transactions. Vol. xxxii.

still higher powers are more successful. But there is one fact, which you must ever bear in mind, in making pathological investigations on the brain, which is this, capillary hyperæmia—the most important of all changes—rapidly disappears after death; if the brain be not examined for two days, you need not be surprised at finding no sign of active disease in the convolutions, after an acute attack of mania. Venous hyperæmia, on the contrary, in dependent points, becomes hourly more aggravated after the death of the patient, and if the blood has become fluid from the evolution of ammoniacal vapours, will produce various discolorations in adjoining structures and tissues which may deceive the inexperienced. Again, the amount of cerebral spinal fluid will be materially increased or diminished, according to the position of the head in relation to the spine, and this has been a source of a thousand blunders; some practitioners actually regarding all fluid beneath the membranes of the brain as abnormal, instead of being what it really is, when not in excess, an essential constituent of the healthy brain. "Softening," again, is frequently the result of a post-mortem change, caused by the contact of fluid, or incipient decomposition: the best test that I know, to determine this point, apart from the time which has elapsed between the time of death and the examination, is to ascertain whether the softening be circumscribed or not, and if it be the former and of a grey colour, and if under the microscope, magnifying 220 diameters, you perceive the compound inflammatory globule of Gluge, or as they have been more recently called, exudation globules, then you may be assured that the softening is the result of vital changes; but if it be diffused over a large extent of surface, if two days have elapsed since the patient died, and if it be destitute of granular cells, simply white in colour, then you may feel certain, that it is a post-mortem result. There is another caution, which is of pre-eminent importance, namely, to be careful that you do not confound the congestion produced by the position of the head, with the congestion induced by abnormal action; by simply leaving the head to depend for a short time below the level of the trunk, you may gorge the posterior sinuses of the head with blood, and produce all the appearance of congestive apoplexy, in the medullary structure; you will at once perceive the vast importance of this fact, in a judicial point of view; it has been forcibly illustrated by Dr. Burrows, in his recent work on the Cerebral Circulation, and as I have not only tested his experiments on the rabbit, but have further ascertained the influence of position in effecting changes in the human subject, I draw your attention to these diagrams with much confidence. These diagrams are in opposition to the previously recorded opinions of the highest authorities in cerebral disease—viz., Abercrombie, Clutterbuck, Copland, and Watson; but I am happy to add, that the latter gentleman has yielded to the force of demonstration, and has recognised the truth and practical value of the facts to which I have been drawing your attention. To return to the necroscopic results in the ninety-four cases, I am enabled to state to you, that the appearances generally observed, after death, in the brains of lunatics, are—in recent cases, inflammation of the arachnoid membrane, and occasionally a deposit of lymph or pus upon its under surface. It is very rarely adherent to the brain. The pia-mater is infiltrated. There are a rosy hue of the vesicular neurine of the convolutions and congestion of the medullary structure of the brain. In chronic cases, there are varying conditions of the calvarium: it is occasionally enormously thickened, but the more frequent change is attenuation, in small circular spots, and sometimes over large spaces about the junction, and at the sides of the frontal and sagittal sutures. Preternatural adhesion of the dura-mater to the calvarium, great opacity, and thickening of the arachnoid membrane, with effusion into and beneath its sac, together with a congested condition of the pia-mater, with effusion of a clear fluid between it and the sulci of the convolutions; the convolutions are frequently marked with small lacunæ or "pits," and are otherwise atrophied; there are varying states of consistence and colour of the "grey matter," mostly pale on the upper stratum, but red, soft, and mammillated in the lower layer of the cortical substance; there is effusion of serum in the lateral ventricles. The choroid flexuses are frequently infiltrated with fluid, and possess simple cysts, varying in size from a millet seed to a currant. With these are various lesions of other organs of the body—the most common being changes in the size and character of the mesentery, displacements of the colon, thickening of the mucous membrane of the bowels, tubercles on the lungs, chronic pneumonia, and cardiac disease. While, in some cases, I have been informed, no lesions whatever have been discovered. This statement I only believe in so far as it is written, no lesion was *discovered.* I have never seen an autopsy of an unequivocal case of *chronic* mania without detecting some cerebral lesion. It would be impossible to enter into the special detail of the ninety-four cases examined, during the time embraced in the table, but I will solicit your attention to the description of one case of acute mania, one of chronic mania, and one of melancholia; and I will then place before you my own views of the pathology of insanity. The case of acute mania fell under my notice in 1847; the patient was forty years of age, and had experienced two attacks of mania, the first of which occurred in the early part of 1845, and was ascribed to disappointment in love; she recovered from this attack, and subsequently married the person to whom she had been previously attached; after having been married eight months, she was deserted by her husband; soon after this, she experienced a miscarriage, accompanied by much hemorrhage, and this was followed by aberration of mind. She was brought to us in March, 1847; she was much emaciated, and her friends informed me that she had not taken food voluntarily for two months. She was at this period very dirty in her habits, and in a deplorable state of melancholia. She was short in stature, and of a nervo-sanguineous temperament. She was frightened at the approach of any person, and held up her hands in an appealing manner, whenever she was spoken to. She seemed to have lost her sense of personal identity, and wandered about the wards, exclaiming that she was not herself, but some other person, and that in consequence of this metempsychosis, some terrible doom would befal her. (I may mention, parenthetically, that this feeling of terror was greatly relieved by morphia.) She improved for a short period, but in November, 1847, an acute attack of mania set in, which defied all treatment, and in nine months after her admission, she died. In the autopsy, the calvarium was found to be normal, the dura-mater healthy, the arachnoid membrane was thickened over the Pacchionian glands, which were few and small, and this membrane was slightly opaque over the cerebellum. The pia-mater was intensely congested, and presented over the face of each convolution quite an arborescent appearance from the injected vessels, which were of a scarlet colour; the most minute artery was plainly discernible, each hair-like ramification standing out in beautiful contrast with the membrane, through which it passed, and the structure upon which it rested. The vesicular neurine of the convolutions was much injected, and had a faint pink hue; the membranes peeled off the brain with the greatest ease. There was no excess of serum within the lateral ventricles, and I should have stated that there was not any in the sac of the arachnoid, or beneath it. The fibrous structure was pale, as contrasted with the peripheral surface: the choroid flexuses were pale, the right was distended, with six or seven cysts, varying in size from a hemp-seed to a horse-bean. On examining the chest, the lungs and heart were found healthy. In the abdomen, the liver was pressed down toward the os ilii, as if from the pressure of stays; but neither it nor the kidneys were diseased; the mucous membrane of the stomach and bowels was healthy, and the uterus and ovaries were in the same condition. I regard this as an interesting and instructive case. It will be observed that the first attack of the malady was induced by nervous shock—that it originated from a moral cause; the second appeared to arise from the disturbance and exhaustion of child-birth and hemorrhage acting upon a mind predisposed to insanity.

The chronic case is selected indiscriminately from a large number. A. B. died on the 29th of May, 1849; she had been in the asylum nearly seventeen years, and had been thirteen years insane when admitted. The calvarium was found normal; the arachnoid membrane was very opaque, thick, and strong, and contained fluid in its sac; the pia-mater was infiltrated with serum; the convolutions of the brain were much atrophied, and the sulci between them looked like so many little rivulets, so full were they of serum; the vesicular neurine was pale and soft. The entire brain weighed two pounds four ounces avoirdupoise. The mitral valve of the heart was thickened, and the left kidney had become converted into a cyst, containing a brownish coloured fluid.

*Melancholia.*—Although melancholia is undoubtedly a variety of mania, as we perceive the symptoms of the one succeeding to the other (as in the cases of S. R. and others) with a precision almost equal to the sequence of day and night, still I have thought it well to refer to it as a distinct form of mental disease, (it being popularly regarded as such,) as it gives me the opportunity to state that displacement of the colon, and a dark colour of the vesicular neurine of the convolutions of the brain are not, as some authors have stated, peculiar to melancholia, and that I know of no lesion which is diagnostic of this form of mental disease; nay, in all the cases which I have examined, the chronic cases of melancholia presented the same appearances which are found in chronic mania. The difference of the symptoms during life being caused mainly (as I think) by the energy of the circulation, or by some character of the blood, rather than by any conspicuous structural change. I shall refer to one of the cases of melancholia, because it possessed an interest peculiar to itself. This case, however, must be regarded not as the type of a form of mental disease called melancholia, but rather as an illustration of one of the *causes* of insanity, to which I shall refer hereafter. The patient was M. G.; she was forty-six years of age, and at the time of her death had been insane ten months. She was the mother of sixteen children, and when admitted into the asylum on Feb. 7, 1848, was suffering from prolapse of the uterus and hepatic disease. She had attempted suicide twice prior to admission, by cutting her throat. The wound inflicted in the last attempt was unhealed when she came to this asylum. She retained the suicidal disposition for five or six weeks after admission, but it disappeared as her health became more impaired by bodily disease. In three months, she became much better in her mind, and left the asylum on leave of absence; she soon, however, returned; from this period she moaned very much, and moved her body to and fro in an agitated manner as she sat in the chair. She always said that "she had been poisoned by quack medicines," "that her bones were rotten," and the like. Her husband said she had never taken quack medicines. She answered all other questions rationally. She died in six months after her admission, from disease of the liver. The autopsy revealed no thickening or distortion of the skull, no opacity or disease of the arachnoid membrane. There was a slight effusion beneath the pia-mater, but so slight that I could not regard it as abnormal. The convolutions of the brain were firm and healthy; their neurine was not discoloured or softened; the fibrous parts of the brain were healthy; there were not more than two drachms of clear fluid in the lateral ventricles—in short, it was a small but healthy brain. The viscera of the chest were healthy. The mucous membrane of the stomach and small intestines were congested, and the liver was extensively diseased, being in that condition which is termed cirrhosis.

It will not serve any useful purpose to enter into descriptive details of individual cases of imbecility and dementia, as they presented, in each instance, appearances similar in kind, but greater in degree, to those which have been detailed to you as characteristic of chronic mania.

*Idiocy.*—One case only of idiocy has fallen under examination in the female department of the asylum. Her brain was very small, weighing only, as I have before stated, one pound six ounces, and having one hemisphere imperfectly formed. Let me remind you that the skulls of some idiots are well formed; there is a congenital idiot in No. 6, Female Ward, with a head of the average female size, and I have known many such.

We have now reached a point in our examination of this most interesting subject, at which we may fairly notice the opinions which have been entertained by various authors respecting the pathology of insanity. They are many and conflicting. We will pass some of them under review, in order to detect the one from the many—to seize the UNITY which must exist somewhere amid all these apparent discrepancies. We will first hear the distinguished Esquirol, who is entitled to the first place among the pathologists of this department of medicine. He says, "The inspection of bodies of lunatics offer numerous varieties as to situation, number, and kind of morbid appearances. The lesions of the encephalon are neither in relation to the disorder of the mind, nor to the maladies complicated with it. Some lunatics whose mental and bodily disease had given suspicion of extensive organic change, have presented but slight lesions of structure in the brain, while others whose symptoms have been less severe, have been the subjects of great and numerous alterations. But what disconcerts all our theories is, that not unfrequently, even in the instances of patients who have passed through all the stages of insanity, and have lived many years under derangement, no organic changes whatever have been traced, either in the brain or its containing membranes." These are startling assertions from one so experienced, but they are greatly modified by the knowledge that the anatomy of the brain was then less perfectly understood than at this moment, and especially by the following expressions, appended to the above observations. "Few bodies of lunatics are *now* examined in which there are not proved to exist at the same time injections or adhesions of the meninges, softenings and tubercles in the brain, serous effusions in the cavities, &c.; while at the period when we made our earliest investigations, we only kept account of obvious and manifest alterations." Greding, after having examined 220 cases, regarded malformation and irregularities in the skull as the chief cause of insanity. Kiesloff, a modern German writer, ascribes the mischief to contractions of the osseous canals at the base of the skull. Bayle regards the inflamed and thickened membranes of the brain as the source of this malady. Guislain says that it is a sanguineous erethism, short of primary inflammation in the brain. Foville, according to Prichard, ascribes the mental affection to organic change in the grey matter of the cerebrum. Cullen writes that it is caused by an irregular distribution of the nervous fluid. Mason Good and Crichton adopt the opinion of Cullen. Nasse regarded the thoracic viscera as at fault. Pinel thought that if it arose from any physical lesion, it was from disordered abdominal viscera. Jacobi shares in this opinion. Weichman ascribes the malady to a contracted colon; Sheppard to disease of the blood. Heinroth, and some others, regard insanity as a purely psychical influence, with which the mere animal organism has nothing to do. The examination of more than 300 cases, and the records of many more, lead me to the belief that each of these writers was correct in the individual instances which came before him, but that he is wrong when the idea is extended to numbers. I feel convinced that jarring discord will ever be found, so long as individuals ascribe this malady to one isolated and individual lesion. The true pathology of insanity (as I believe) is recorded in the following table; but the limits of this lecture will prohibit my entering into the proofs of each position; they can, however, be fully substantiated by facts in my possession. I might have been more precise than I have been; thus you will perceive that I state—

### INSANITY MAY BE PRODUCED

*PRIMARILY*

*A.*—By some change in the Brain itself, induced by moral (psychical) causes—*Joy, Grief, Fear, Anxiety, Shame, &c., Intense and prolonged Intellectual exertion.*

*B.*—By similar change or changes induced by physical (somatic) causes in the Brain—*e. g. Impaired nutrition. Irregular Development of the Hemispheres.*

*SECONDARILY.*

*A.*—By malformation, or disease of the Bones of the Skull.

*B.*—By changes in the Membranes of the Brain.

*C.*—By changes in the Cerebral Circulation—*e. g. Deficiency of blood through a mechanical closure of the Arteries from Atheromatous deposits, or by Arteries otherwise diseased. By retardation in the return of venous blood from any cause, mechanical or vital. By an excess of blood, or its too active circulation; or the converse of these from various conditions of the heart, and other causes.*

*D.*—By impoverished Blood (impaired nutrition). By Blood containing other than its healthy constituents—*e. g. Blood containing Alcohol, "Hachish," Lead, &c.*

*E.*—By defect or disease of the Organs of Sense.

*F.*—By diseases of the Assimilating Organs.—Primary—*Unhealthy Chyle.* Secondary—*Arrest of Secretions.* See above Blood diseases.

*G.*—By sympathy of the Brain with the irritation of remote Structures and Organs—*e. g. The Skin, Mucous Membranes, Heart, Lungs, Stomach, Liver, Kidney, Bladder, Testes, Ovaries, Uterus.*

*** Each and all of these causes act by producing functional disturbance, or structural change, in the vesicular neurine (grey matter) of THE ENCEPHALON.

---

IDIOCY—Vesicular Neurine of the Brain, congenitally defective;—or changed in its character by Convulsive, or other Diseases in Infancy.

---

Now, I have designedly used the word "encephalon," because there are some portions of the base of the brain, and of the cerebellum, the pathology of which is not as yet understood, and of which I entertain some notions, not yet fully matured for publication. I wish, however, to add, that there are many persons whom it is quite necessary to detain in Lunatic Asylums, whose brains are not *diseased*—in the strict sense of that word, nor are they sympathetically affected by the lesions of remote organs, or irritated by poisoned blood. The organs of the whole system are physically healthy, and their functions are carried on in accordance with health; but they have an imperfectly developed brain—the instinctive emotions predominate so largely over the higher faculties of the mind, as to render their actions quite incompatible with the well-being and comfort of society. In such individuals, were they to die suddenly from some accident, there would be no disease of the calvarium, no thickening of the meninges, no structural change in the brain to be seen after death. These persons are prone to insanity of a violent and persistent form, but there is a lengthened period of their existence in which there is no somatic disorder—the individual, in fact, is living the life of an animal, while he is surrounded by the conventionalities, the laws, the privileges, and the requirements of a more exalted state of being. I regard M. C., in No. 6 ward, as an elevated example of this class of individuals.

There is another class of persons, the very opposite of these, who may be the subjects of various hallucinations, and yet be free from any active or sympathetic disturbance of the brain. There is an attribute of the human mind called imagination; it is variously possessed by different individuals. There are those so cold, so passionless, so destitute of the ideal faculty—so abstractedly intellectual, so purely scientific, that, as Wordsworth says, they "would peep and botanize upon their mother's grave." It is not to these that I now refer. "What do you see, sir?" said Fuseli one day to a student, who, with his pencil in his hand, and his drawing before him, was gazing into vacancy. 'Nothing, sir," was the answer. "Nothing, young man!" said Fuseli, emphatically; "then, I tell you, that you ought to *see something*—you ought to see distinctly the true image of what you are trying to draw. I see the vision of all I paint, and I wish to Heaven I could paint up to what I saw!" It is a rich endowment, this imagination. It clothes the landscape with beauty, and spreads refinement and grace over all things. Its pleasures have been described in a glorious poem by a member of our own profession: its utility has been proved by the sublime discoveries of Kepler, and the beautiful theories, and splendid results of Sir Humphry Davy. Endowed with this faculty, we may with Rogers clothe the past with the soft hues of memory, or, with Campbell, illumine the future with the sunshine of hope. By its power, a Shakspeare can startle the mind with the hideousness of a Caliban, the guilt of a Macbeth, and the treachery of an Iago—or fill it with pure delight by the creation of such beings as Ariel, Miranda, and Cordelia;—in short, passing the confines of earth, it can, with Dante, penetrate the gloom and the darkness of the prison-house of souls, or, with Milton, hear the song of angels, and see them prostrate their crowns of amethyst and gold in adoring ecstasy, before the throne of the Omnipotent. All this may be done with impunity, when the perceptive faculties are healthy, and the imagination is modified, directed, and controlled, by a sound judgment; but when the perception is weak, and the judgment not strong, an excess of this ideal faculty leads to eccentricities of conduct—to Utopian schemes, and wild visions verging on insanity. To such minds do I refer—thus, the painter Blake, lived in an imaginary world, and was guilty of freaks, which, had he not possessed a gentle, kind, heroic wife, might have confined him for life within the walls of a lunatic asylum. Allan Cunningham, in his charming "Biographies of British Painters," relates, "visions, such as are said to arise in the sight of those who indulge in opium, were frequently present to Blake; nevertheless, he sometimes desired to see a spirit in vain. 'For many years,' said he, 'I longed to see Satan—I never could believe that he was the vulgar fiend which our legends represent him—I imagined him a classic spirit, such as he appeared to him of Uz, with some of his original splendour about him. At last, I saw him. I was going up stairs in the dark, when suddenly a light came streaming amongst my feet; I turned round, and there he was looking fiercely at me through the iron grating of my staircase window. I called for my things; Katharine thought the fit of song was on me, and brought me pen and ink. I said, 'Hush! never mind,—this will do;—as he appeared, so I drew him —there he is!' Upon this, Blake took out a piece of paper, with a grated window scratched on it, while through the bars gazed the most frightful phantom that ever man imagined. Its eyes were large, and like live coals, its teeth as long as those of a harrow, and the claws seemed such as might appear in the distempered dream of a clerk in the Herald's Office. 'It is the Gothic fiend of our legends,' said Blake—'all else are apocryphal.'" Cases of hallucination, occurring once only in life, and ever after influencing the conduct of the individual, are occasionally met with, in whom there is probably no appreciable physical lesion, but where, from some temporary excitement, the mind has become impressed with the objective reality of its own imaginings—although not habituated to this, from special organization as men, like Blake; or, where the senses have been so acted upon by diffused, or imperfectly defined objects, as to impress with false notions the perceptive faculties, and through them the reflective powers of the mind. In all ages, in all climes, in all churches, there have been enthusiasts whose fervid imaginations have thus deceived their senses. Paganism abounds with such tales, and I for one believe, that the great founder of Islamism was as often self-deceived as deceiving—that, in those frequent retirements at the cave of Mount Hara, where he went to muse on the hollowness of the idolatry around him, and the reality and greatness of that Allah whom he adored, his imagination and feelings became so excited, that his senses were no longer faithful, and that visions were seen, and voices heard, which he enforced so powerfully afterwards, both by tongue and sword. And we know that, in the venerable church to which we owe so much, for preserving alive the elements of freedom, of science, of poetry and art, during the long dark night of barbaric feudalism, there were many such self-deceived enthusiasts—not even the genius of Ignatius Loyola was exempt, for he tells us, that in the cavern of Manresa, after long fastings, maceration, and penance, as "he lay awake in his bed at night, he saw plainly, with his *bodily eyes*, the blessed Virgin appearing to him with the Holy Infant in her arms, both regarding him with looks of benign complacency and encouragement." Again, the distinguished monk of Saxony, writing in the lone room at Wartburg, and worn by fatigue and study, imagined that Satan had come in person to confront him, and to destroy his labours. He seized upon the inkstand, and hurled it at the fiend. It was shivered on the opposite wall. The spot remains to testify to the temporary hallucination of the man, at the same time to proclaim to all generations that no power of earth or hell could shake for a moment the great soul of Luther, or deter it from the accomplishment of that mission which he believed it his duty to fulfil.

The Giant of the Hartz Mountains, or, better still, the white gravestones which in a dark and gusty night have appeared as walking spectres, steeple-high, to the terrified rustic, are illustrations of my meaning in the second particular. The hallucinations excited by such feelings, and such objects, although accompanied, as all thought probably is, by molecular changes in the brain, would leave no structural lesion which the eye could detect, unless followed by more confirmed insanity.

The causes arranged in the second group act only so far as they influence the first. Moral causes, according to Pinel, prevail over the physical; and Esquirol, in a memoir presented to the Society of Medicine, states that cases of madness, occasioned by moral causes, are to those arising from physical causes, as 4 to 1. They are placed, you will perceive, in Class A of the primary group.

The experience of Esquirol and Pinel accords with that of this institution, in assigning the preponderance to moral causes. I am, however, quite convinced that in the majority of cases of insanity, the *cause* will be found to be both moral and physical—*i. e.*, there must be previous physical weakness in the brain for the moral shock to end in permanent lunacy. In estimating moral shock, we have no accurate standard for our guidance. The hide of the rhinoceros, and the apex of the elephant's trunk, differ not more widely in their susceptibility to impressions than the minds of various men.

"Chords that vibrate sweetest pleasure,
Thrill the deepest notes of woe."

But the influence of impressions are different at different periods, and some minds are more sensitive to one feeling than to another; thus, on February the 28th, the same afternoon in which Dr. Conolly, with great eloquence, was addressing the members of the College of Physicians on the causes of insanity, there was tossing in the Straits of Dover a ship called the "Floridian," with 200 emigrants on board. It was a most tempestuous day. As the evening drew in, the storm at sea became more fierce; the snow fell fast, darkness covered the deep, and the vessel was in great peril. From some cause, during the night, the officers lost their reckoning—the ship was dashed against the Essex coast, and off Harwich became a wreck. While she was falling to pieces, two sailors and an emigrant lashed themselves to the masts, and upon these were floated off amid the breakers. During that long night, and the following day, they were tossed to and fro upon the surging waters; and when at last they were rescued from their perilous position, two of them were well-nigh dead from exhaustion—nay, the emigrant was exhausted in a similar manner; but, while warmth, rest, and safety brought back reason, health, and vigour to his companions, they aroused in him the ravings of insanity—thus, in two men out of three, fright, danger, and cold, produced bodily exhaustion alone—while, in the third, perhaps from *greater* fright, they ended in madness. From him the impress of that fearful night may never pass away—darkness, confusion, and fear may be his for ever.

To revert to the pathological synopsis : moral causes are placed in Class A of the primary group; while blows on the head (like to that which produced epilepsy, and subsequently insanity in J. G.), "sunstrokes," idiopathic inflammation of the brain, and the like, fall into Class B of the same order of causes.

---

There are several skulls on the table, which illustrate the various changes which the bones of the head sometimes undergo in long-continued insanity. In one, you perceive that the osseous structure has become much thickened and spongy: viewed through the microscope, it is one mass of perforations, and I have been unable to detect in any portion of it a single well-formed bone cell.

That specimen is three-quarters of an inch in thickness, and forms a marked contrast to this cranium, which in many parts is not thicker than cartridge paper: you observe, that it is diaphanous over a large portion of the frontal and parietal bones; it is as hard and brittle as the other specimen was soft and spongy: viewed through a quarter-inch lens, it will be found to possess a large number of "bone-cells," and the Haversian canals are exceedingly minute. These cases are similar to many which Greding examined; and without now discussing the matter as to whether these changes are causes, or coincidences, or consequences, we admit such malformations and diseases as occasional causes of insanity, and group them in class A of the secondary series.

The numerous cases of Bayle, and those reported by Dr. Webster and others, where thickened and inflamed meninges were the only appreciable lesions to be found after death, are allocated in class B.

I have so frequently found all the arteries supplying the brain in a diseased condition, from atheromatous deposits, and other changes, that I recognise such lesions as one of the causes of mental derangement; this group C, may also receive the cases referred to by Kiesloff, as arising from contraction of the osseous canals at the base of the cranium.

Among the causes arranged in the secondary group, few are more important than those placed in class D. A systematic study of mental diseases has been carried on in England for much too short a period to enable us to give precise and definite details of the specific character of the blood, by which insanity is produced. Our facts are too few, and organic chemistry itself too imperfect for such a desirable consummation, beside which, the subtle essence, which may be sufficient to rouse the nervous system into the wildest action, may have entered into new combination, by the very process which had been resorted to for discovering its presence, especially when the nature of the agent is wholly unknown, and no clue is given to the chemist by which he may trace out its presence, and display its character. It is, however, quite certain that each organ has, as it were, an idiosyncracy of its own, by which it is more susceptible of the influence of one poison than of another—*e. g.*, the liver and the salivary glands to calomel, the spinal system to strychnia, the brain to opium, and the like. A remarkable instance of the effects which some drugs produce upon the senses and the mind, came under my observation in the summer of 1844. A lady, (the wife of a captain in the navy,) who was suffering from spasm about the uterus, had two grains of the extract of Indian hemp prescribed for her by the physician in attendance; soon after taking it, she fell asleep; between two and three hours after, she awoke, with an excruciating pain on the top of her head, which was followed speedily by hallucinations of a frightful character. She recognised all the friends who approached her, and spoke to them in an entreating manner about the spectres around her bed. She occasionally screamed with horror; at other moments, she was wanton in her expressions and gestures. She saw demons, hideous in form and threatening in aspect, and for two hours she was full of terror. As these hallucinations passed away, another, of a different character, remained: she felt herself to be in the presence of a handsome person, listening, with pleasure, to suggestions which she afterwards contemplated with much remorse. She was a very pure-minded, religious person, and subsequently informed me, that frightful as were the spectres, the feelings which came on afterwards were so abhorrent to her, "so sinful," that she hoped the drug would never be administered to her again. It was administered again, clandestinely, by her medical attendant, who could not believe that such effects were produced by the drug, but regarded them as the offspring of hysteria. Precisely the same results ensued, and for six hours this lady was decidedly insane. I watched the whole of these symptoms throughout their duration, and am confident that they were neither feigned nor the offspring of hysteria, but purely the result of the drug; and the eminent physician who prescribed it, now shared in the same opinion. The hallucinations, in the latter stages of the drug's operation, may have assumed the forms they did from the pre-existing disease about the ovaries, as the delusions of E. J——, described in my former lecture, took their rise apparently from disease about the abdomen; and nothing is more common than for us to discover bodily ailments in the first instance, by the peculiar "delusions" of the patients. The insanity which is dependent upon some poison circulating in the blood, is frequently of short duration, as in the case just detailed to you—(perhaps "delirium"*—is a more strict appellation than insanity for such cases, but it obviously differs from the latter disease only in the extent of its duration)—the poison being carried out of the system by some of the emunctories of the body. The duration of the disease will vary in proportion to the success of remedial measures. The miasma-poison, whatever it may be, which produces intermittent fever, continues, in some persons, to exert its baneful influence for many weeks, and even months, while in others, it rapidly disappears under the use of blue pill, emetics, and quinine; and in like manner, will the duration of such cases of mania be dependent on the principles of treatment. The comparison between these blood poisons reminds me, that Sydenham has recorded that mania frequently supervened upon the intermittent fever which prevailed between the years 1661 to 1664. I believe that in some of the cases of "suppressio mensium," there is a morbid condition of the blood; there is retained in the blood that which contaminates the system, in the same manner as urea may do when the renal function is suspended. It has been long observed by practitioners, who have had much to do with female diseases, that there is some connexion between unhealthy uterine secretions and rheumatism, and I have no doubt that there is a close connexion, in some cases, between these and insanity. In by far

* The ready manner in which she recognised her friends at the bedside, distinguished it from delirium.

the greater number, this important function acts simply by relieving the brain of congestion, in the same manner as the hemorrhoidal flux in males; but in others, there is a depurating agency effected by it; near to the catamenial period, some chronic lunatics become fierce, and the melancholic suicidal. The cases of insanity in which a morbid poison has been generated in the body and absorbed by the blood, are distinct from those which arise from defective blood—blood wanting in fibrine—and which are characterized by a general debility of the muscular system at the same time. These cases are more common, perhaps, in agricultural than commercial districts. Some of these patients are irritable and noisy, but a very common feature in this form of the malady is extreme taciturnity, or they speak in monosyllables, are indifferent to their dress and surrounding things; others are insensible to, or careless of danger, and make feeble efforts at self-destruction, their attempts are not concealed or vigorous, and seem to arise from no definite purpose—*e. g.*, they attempt to go down a staircase regardless of the steps, or to pass through a window of an upper story; but all their actions are dream-like and feeble.

It may occur to you that my table embraces almost every disease,—it does so; and there is no disease mentioned in it which has not been the precursor of insanity.

Can so many lesions produce one distinct effect? I answer, *Most certainly, in parties predisposed to mental disease.* We meet with the same phenomena in every malady. The lungs are commonly regarded as the seat of the respiratory function, and yet disease in the brain will affect respiration. Pressure upon the pneumogastric nerve at its origin, or in its course, will diminish or disturb this function as completely as an irritant upon the lung itself. I wish to place the diseases of the brain upon the same scientific basis as the diseases of other organs; in other words, I am anxious to prove, that the pathology of insanity is not more paradoxical than is the pathology of respiration, or any other important function. And I am sure that the especial lesions of the brain which produce insanity, may be demonstrated with as much accuracy as lesions which produce disturbance and disease in any other intricate structure. The above table contains the germs, the nucleus of the truth in this matter. I feel that I have reconciled the discrepancies of all authors on this subject, and that my humble labours have not been in vain. But to return. Can so many various lesions produce one distinct effect? I repeat, most certainly. Bear with me, while I carry out the analogy to which I have already referred. Injuries or diseases of the cranium may disturb the intellect—disease, or injury of the ribs, the bony covering of the lung, may disturb respiration. Diseases of the dura mater, the periosteum of the skull may derange the understanding—periostitis of the ribs may affect the breathing; inflammation of the arachnoid, or effusion of serum or lymph from its surface, may lead on to insanity; inflammation of the pleuræ, and its consequent exudations, embarrass respiration; diseases of the arteries produce parallel results in both cases; and different poisons conveyed by the blood affect both organs in an analogous manner. In neither of the structures of the lung named is the function of respiration especially located, nor is any one of the parts of the brain enumerated, the precise seat of intelligence; but all these lesions operate upon these two respective functions, in a corresponding ratio to their encroachments upon the ultimate ramifications of the air-tubes in the one case, and the vesicular neurine of the convolutions in the other. *In every case of insanity, there is irritation, or disease of the grey matter in the encephalon*—the especial *character* of this disease may be different in each case, while *irritation* of this structure may arise from simple sympathy with remote organs. In very rare cases, the disease may be limited to simple erythema of the convolutions; and where this is strictly *idiopathic,* and *isolated,* I believe no physical disorder would be manifested in other organs of the body; just as in simple arachnitis, a most rare disease, there would be no disorder of the intellect. Dr. Hodgkin has described such cases; and the case of S., referred to in my first lecture, goes to show that insanity may exist for a long time, without *necessarily* involving this membrane. You will perceive by this accurate drawing, that the vesicular neurine consists of two distinct portions, separated by a very fine delicate white line. This line of demarcation has doubtless an important purpose, and these two parts are variously affected in diseases, as shown by variety in their respective colour and consistence in some cases. The upper portion, under the microscope, is formed of such simple elements, such minute granular masses, that I have been unable to detect any marked histological change in its structure, under chronic disease, except that the granules are more diffused, and, as it were, separated from each other by a thin homogeneous fluid. In recent cases the change in its blood-vessels and its consequent colour is well marked. In chronic cases of dementia, of an aggravated form, the nerve-vesicles in the lower planes of the grey matter of the convolutions have appeared to me to be broken up and disintegrated; and by a simple lens, when the upper plane of grey matter is carefully removed, this lower plane is seen to be rough and mammillated.

Time forbids my enlarging upon this absorbing theme. No one can more deeply feel that this lecture on the pathology of insanity has been defective and imperfect, than I do. If, however, it has awakened an interest in your minds, so as to induce you to study the malady, my purpose has been fulfilled. I want to see young, and ardent, and honest minds engaged in this pursuit. It is a field in which "the harvest is indeed plenteous, and the labourers are few." The malady needs investigation, for it seems to be marching onwards with fearful strides. Its consequences are tremendous. It renders useless the strength of the peasant, the ingenuity of the mechanic, the skill of the artisan, the genius of the painter and the poet, the learning of the scholar, the wisdom of the statesman, and the patriotism of the monarch; its influence stretches from the cottage to the throne. Like Death itself,—

> "Æquo pulsat pede, pauperum, tabernas

> Regum que turres."

This being so, it becomes all-important to investigate its nature. In what, then, does the disease *essentially* consist? what is the especial and "*ultimate*" change in the convolutions of the brain, by which this malady is produced? This question cannot be answered at the present time. Although there *must be* some one *elementary* change, upon which insanity is dependent, it is doubtful whether it will ever be demonstrated: there will occur a multiplicity of visible changes in that structure, by any of which insanity may be caused through embracing the momentary one; and these alone will be demonstrated by future research. Herein insanity differs from no other disease. The boundaries of our wisdom are quickly reached. With all our boasted acquirements, we stop at the border-land of actual knowledge. It is the same in physical science, apart from life. We see a stone fall to the earth, and we try to explain it, by ascribing it to the unerring law of gravitation, but what is this? The difficulty becomes tenfold greater when life is superadded; for, depend upon it, the mysteries of sense and thought will never be unravelled by any comparison with merely physical agencies. Let us not hide our ignorance in a cloud of pompous words, and then flatter ourselves that we are wise. We are living in a time when the vital endowments of organs are overlooked, and persons begin to imagine that electricity may produce a monad, which, in the progress of ages, may become a man! Creation and life are mysteries higher than can be reached by human philosophy. Let us study the wondrous endowments of organs, and the obvious conditions upon which their healthy manifestations depend, leaving nicer subtleties to vainer men, and contenting ourselves with the divine tradition—

